# Role of Intestinal Alkaline Phosphatase in Innate Immunity

**DOI:** 10.3390/biom11121784

**Published:** 2021-11-29

**Authors:** Sudha B. Singh, Henry C. Lin

**Affiliations:** 1 Biomedical Research Institute of New Mexico, Albuquerque, NM 87108, USA; sbsingh14@salud.unm.edu; 2 Medicine Service, New Mexico VA Health Care System, Albuquerque, NM 87108, USA; 3 Division of Gastroenterology and Hepatology, Department of Internal Medicine, University of New Mexico, Albuquerque, NM 87131, USA

**Keywords:** intestinal alkaline phosphatase, LPS, autophagy, barrier function, inflammation, gut microbes

## Abstract

Intestinal alkaline phosphatase (IAP) is a multi-functional protein that has been demonstrated to primarily protect the gut. The role of IAP in maintaining intestinal homeostasis is underscored by the observation that IAP expression is defective in many gastrointestinal-related disorders such as inflammatory bowel disease IBD, necrotizing enterocolitis, and metabolic syndrome and that exogenous IAP supplementation improves the outcomes associated with these disorders. Additionally, studies using transgenic IAP-knock out (IAP-KO) mouse models further support the importance of the defensive role of IAP in the intestine. Supplementation of exogenous IAP and cellular overexpression of IAP have also been used in vitro to dissect out the downstream mechanisms of this protein in mammalian cell lines. Some of the innate immune functions of IAP include lipopolysaccharide (LPS) detoxification, protection of gut barrier integrity, regulation of gut microbial communities and its anti-inflammatory roles. A novel function of IAP recently identified is the induction of autophagy. Due to its critical role in the gut physiology and its excellent safety profile, IAP has been used in phase 2a clinical trials for treating conditions such as sepsis-associated acute kidney injury. Many excellent reviews discuss the role of IAP in physiology and pathophysiology and here we extend these to include recent updates on this important host defense protein and discuss its role in innate immunity via its effects on bacteria as well as on host cells. We will also discuss the relationship between IAP and autophagy and how these two pathways may act in concert to protect the gut.

## 1. Introduction

Intestinal alkaline phosphatase (IAP) is a member of the alkaline phosphatase family and is one of the four types known to exist in humans: tissue non-specific alkaline phosphatase (TNAP), placental alkaline phosphatase (PLAP), germ cell alkaline phosphatase (GCALP), and intestinal alkaline phosphatase (IAP). IAP is produced by enterocytes in the small intestine and is secreted into the lumen, blood, and stool. IAP deficiency has been linked to plethora of conditions such as IBD, type 2 diabetes, ischemic heart disease, aging, necrotizing enterocolitis, and metabolic syndrome, among others [1,2,3,4].Supplementation with IAP has been tested in clinical and pre-clinical studies for its protective role in various diseases. Many functions thus far have been ascribed to IAP: (1) IAP detoxifies lipopolysaccharide (LPS) [5], which is responsible for causing gut permeability and inflammation; (2) IAP dephosphorylates pro-inflammatory nucleotides such as ATP and UDP [6]; (3) IAP regulates bicarbonate secretion and maintains duodenal pH [7]; (4) IAP protects the intestinal epithelial barrier by regulating the level of tight junction proteins such as occludins, claudins, and zonula occludens [8]; (5) IAP regulates gut microbial communities [9]; (6) IAP appears to positively regulate antimicrobial proteins such as lysozymes that control the bacterial numbers in the intestine [10]; (7) IAP induces autophagy [10], a vital innate immune pathway known for its role in barrier function, inflammation, and its antimicrobial functions; (8) Some reports also suggest a positive correlation between IAP and mucins that make up the mucus layer in the intestine [11,12]. In this review, we discuss in detail current advances in understanding the innate immune functions of IAP that serve to protect the intestinal epithelium. Furthermore, we will discuss the relationship between IAP and autophagy and how these two important innate immune pathways may orchestrate downstream events to defend the gut at multiple levels.

## 2. IAP and Barrier Function

Integrity of the intestinal epithelial barrier is paramount to health [13]. Dysfunction of barrier integrity leads to increased intestinal permeability (leaky gut) which has been linked to many diseases such as IBD, neurodegenerative diseases, and metabolic syndrome [14,15]. Barrier integrity is maintained through the coordinated action of a number of mechanisms that together comprise the first line of defense. These include: (1) the physical epithelial barrier constructed by tight junction proteins (TJPs), adherent junction proteins (AJPs), and desmosomes which prevents paracellular flux and microbial translocation; (2) the mucus layer, comprised of various O-linked glycoproteins called mucins, which acts as a partition between the bacteria and the epithelium and prevents their translocation into the epithelium; (3) antimicrobial peptides and proteins such as lysozyme that kill microbes, thus controlling their numbers in the intestinal lumen; (4) IAP, which protects the barrier directly from harmful effects of LPS and indirectly by orchestrating the other three mechanisms of the first line of defense. 

### 2.1. IAP Protects the Intestinal Barrier Integrity in Various Disease Models

IAP has been demonstrated to protect the gut barrier function in many animal models of diseases. The role of IAP was demonstrated in mouse models of common bile duct ligation (CBDL) and carbon tetrachloride (CCl_4_) models of liver fibrosis using oral supplementation of IAP as well as IAP-KO transgenic mice [16]. IAP rescued the decreased levels of tight junction proteins (TJPs) and decreased the plasma concentration of FITC-dextran, a marker of leaking across the intestinal barrier. In burn mouse models [17], increased FITC -dextran in blood was observed in IAP-KO mice when compared to WT mice. Moreover, burn wound infection-induced death was accelerated in IAP-KO mice compared to control mice. Conversely, oral supplementation of IAP attenuated burn-induced gut permeability by lowering the plasma levels of FITC-dextran and by increasing TJPs ZO-1 and claudin-1. IAP supplementation also attenuated burn-induced inflammation and improved overall survival of mice. Treatment with oral IAP was found to attenuate alcohol-induced hepatosteatosis and increased gut permeability, endotoxemia, and inflammation in mice [18]. 

A study by Kuhn et al. [4] also demonstrated the role of IAP in aging. Aging mice had lower levels of IAP which correlated with increased intestinal permeability, increased endotoxemia and inflammation, and changes in gut microbiome. Age-related outcomes were even more pronounced in IAP-KO mice. Adding exogenous IAP to aging mice attenuated age-related increased intestinal permeability, inflammation, prevented changes in gut microbial profiles, and increased the overall life span of mice. IAP also improved the physical performance and life span of Drosophila in the same study. In a mouse model of peritonitis, IAP supplementation reduced intestinal permeability and suppressed gut bacterial translocation into the blood stream, liver, and lung [19]. In another study, IAP reduced the magnitude of increased permeability in the small intestine of a cecal ligation and puncture (CLP) mouse model [20].

Interestingly, the role of IAP in protecting the gut barrier in extra-intestinal disorders has also been reported. Recently, the role of IAP in Western diet (WD)-induced atherosclerosis was examined using a transgenic hyperlipidemic Ldlr^−/−^ mouse model overexpressing intestine-specific human chimeric IAP (Ldlr^−/−^IAP^Tg^) [11]. It was found that IAP attenuated WD-induced reduction in colonic mucus layer, lowered WD-induced LPS levels in plasma, and reduced plasma and hepatic lipids, among other WD-induced atherosclerosis-related outcomes. Intestine-specific transgenic overexpression of IAP in IAP^Tg^ mice improved intestinal barrier function as measured by FITC-dextran levels in plasma as well as reversed the reduction in fecal zonulin in WD-fed mice [21]. IAP supplementation also attenuated neuroinflammation in a mice model of heart failure (HF) including reducing intestinal barrier permeability, decreasing plasma LPS levels, and increasing protein levels of tight junction proteins in HF mice [22]. Treatment with L-phenylalanine, an inhibitor of IAP activity, abrogated the protective effects of IAP in HF mice. It was found that maternal treatment using oral IAP attenuated high fat diet (HFD)-induced autism-like cognitive disorder in offsprings [23]. However, whether the leaky gut phenotype was observed in such offspring and whether IAP reversed it is not known. Thus, many studies have highlighted the importance of IAP in attenuating adverse outcomes associated with a diverse number of diseases. Detailed mechanisms of how IAP interacts with host cellular pathways in these disease models need to be explored further. One way by which IAP protects the barrier is by regulating tight junction proteins (TJPs) that make up the physical barrier and the microbial community that resides in the gut. In the following section, we will describe these roles of IAP and how they may regulate the innate immune response via autophagy in more detail.

### 2.2. IAP and Tight Junction Proteins (TJPs)

One of the underlying mechanisms responsible for downstream effects of IAP on epithelial barrier function is the regulation of tight junction proteins (TJPs) by IAP. This has been reported in many studies. Mouse embryonic fibroblasts derived from IAP-KO mice had lower levels of TJPs ZO-1, ZO-2, and occludin. Consistent with a loss of barrier integrity in the whole animal, IAP-KO mice demonstrated increased gut permeability and decreased levels of TJPs ZO-1, ZO-2, occludin, and claudin 1 when compared to WT mice [24]. IAP supplementation was shown to prevent alcohol-induced gut barrier dysfunction and TJP loss [18]. In mouse models of burn injury, exogenous supplementation of IAP reversed the loss of ZO-1 and claudin-1 [17]. Human colon cells Caco2 and T84 cells overexpressing IAP had elevated levels of TJPs [8]. In Caco2 cells, treatment with exogenous IAP increased transepithelial electrical resistance (TEER) and decreased claudin-2 (a mediator of leaky gut) expression [19]. Thus, several studies have provided evidence that the protective effect of IAP on barrier integrity is via its effect on TJPs. How IAP regulates TJP expression and distribution remains to be explored. Further mechanistic studies are needed to dissect out the direct or indirect effect of IAP on TJPs. In addition, it remains to be determined whether IAP affects other junction-associated proteins such as adherent junctional proteins (AJPs), desmosomes, and coxsackie virus B adenovirus receptors (CARs) [25] to maintain epithelial layer integrity.

## 3. IAP, Inflammation and TLR4

IAP is well known for its anti-inflammatory role in the gut, mainly via its direct detoxification of pro-inflammatory molecules such as LPS and nucleotides such as adenosine triphosphate (ATP) and uridine triphosphate (UDP) [5]. The nucleotide UDP is the specific ligand for the P2Y_6_ pyrimidinergic receptor which has been directly implicated in immune function and intestinal inflammation [26]. On the other hand, ATP has a more direct role in inhibiting bacterial growth in vitro and in vivo by affecting pH, thus contributing to gut bacterial dysbiosis [6].

IAP also has an indirect role in maintaining barrier function by inhibiting proinflammatory molecules such as LPS and cytokines including tumor necrosis factor-alpha (TNF-α) and IL-1β, which disrupt barrier function. Danielak et al. showed that supplementation of IAP in combination with voluntary exercise caused a reduced expression of proinflammatory cytokines TNF-α, IL-1 β, and IL-6 in a colitis mouse model fed with standard diet (SD) or high-fat diet (HFD) [27]. In another study, oral IAP reduced TNF-α and IL-6 in brains of mice mode of heart failure [22]. IAP ameliorated colitis in mice and inhibited LPS-induced IL-6 and TNF-α production [28]. An indirect anti-inflammatory role of IAP is also evident by our own observation that showed autophagy-dependent anti-inflammatory function of IAP [10] where IAP inhibited LPS-induced inflammatory NF κB subunit p65 phosphorylation as well as IL-1β gene expression in macrophages. 

The protective role of IAP in inflammation occurs in a toll-like receptor 4 (TLR4)-dependent manner. TLR4 belongs to the family of toll-like receptors (TLRs) that are activated by various stimuli expressing pathogen-associated molecular patterns (PAMPs). These PAMPs are expressed by bacteria and viruses as well as fungi [29]. TLR4 receptor is activated by lipopolysaccharide (LPS), an endotoxin secreted by Gram-negative bacteria responsible for inflammation [30]. It was found that IAP protected against colitis in mice in a TLR4-dependent manner [28]. In the same study, in peritoneal macrophages derived from the wild-type (WT) but not in TLR4-deficient (TLR4^−/−^) C57BL/6 mice, IAP inhibited the LPS-induced tumor necrosis factor-alpha (TNF-α) and interleukin-6 (IL-6). Similarly, IAP rescued the outcomes associated with CCl4 and CBDL-induced liver fibrosis in WT and IAP-KO mice but not in TLR4^−/−^ KO mice [16]. In our own study, we showed that IAP induced autophagy in a TLR4-dependent manner [10]. Moreover, TLR4 gene expression was also upregulated in small intestine in mice given IAP in drinking water. All these studies have indicated the requirement of TLR4 for IAP to mediate its effects. As TLR stimulation negatively affects tight junction proteins and barrier integrity [31,32], it is imperative to study whether and how IAP regulates TJPs in a TLR4-dependent manner. While IAP directly binds to LPS, a ligand for TLR4, how it mediates its direct effects via TLR4 is a subject of future investigation. Understanding of the relationship between IAP and TLR4 will shed further light on the function of IAP and may be helpful in identifying novel therapeutic targets.

## 4. Role of IAP in Maintaining Gut Microbial Homoeostasis

The gut microbiome is an integral part of host physiology and pathophysiology. Intestinal microbial dysbiosis has been found to be associated with many disorders such as inflammatory bowel disease (IBD), irritable bowel (IBS), infectious diseases, autism, cancer, metabolic syndrome, and even aging [33,34]. While gut microbes have a profound effect on host physiology, host factors such as IAP also play a critical role in shaping and controlling the resident community of gut bacteria. It has been found that there were significantly fewer bacteria and different type of aerobic and anaerobic bacteria in IAP-KO mice when compared to the WT mice [35]. In another study, bacterial growth was shown to be inhibited in the jejunal loop in IAP-KO mice and intestinal juice obtained from IAP-KO mice showed reduced growth-promoting effects on *E. coli* when compared to growth of these bacteria in the presence of WT luminal juice [6]. Growth-promoting effects of IAP on bacteria were found to be due to the inhibition of luminal ATP by IAP by inhibiting the alkaline pH caused by ATP [36,37]. On one hand, IAP promotes the growth of commensal bacteria; on the other hand, IAP has an antimicrobial effect on gut bacterial population associated with disease. De Lisle et al. showed that treatment with exogenous IAP caused a decrease in the bacterial density in small intestinal bacterial overgrowth (SIBO) associated with cystic fibrosis in a mouse model [38]. IAP also protects against pathogenic bacteria and prevents bacterial translocation. IAP supplementation was shown to protect mice from *Clostridium. difficile* and *S. typhimurium* infections during antibiotic treatment [35,39]. In a study by Yang et al., infection of mice with *S. typhimurium* lowered the levels of IAP in the intestine, increased the amount of LPS in the intestinal contents, and increased barrier permeability [40]. It was shown the bacteria affected IAP function by inducing neuraminidase enzyme to cause desialyation of IAP in a TLR4-dependent manner. Oral supplementation of IAP reversed these effects of *S. typhimurium* on the host.

IAP may also control bacterial populations indirectly by upregulating proteins such as lysozyme. Lysozyme is an antimicrobial protein which shapes up the gut microbial composition in addition to eliminating pathogens [41]. It was shown by our recent study that lysozyme gene expression was increased in the small intestine in mice given oral supplementation of IAP [10]. While the exact mechanism by which IAP influences lysozyme is not known, one possible candidate mechanism is IAP-induced autophagy, a well-known antimicrobial degradative pathway [42] which is a positive regulator of lysozyme [43].

## 5. IAP and Autophagy 

Our recent study showed that IAP induced autophagy in macrophages and in epithelial cells [10], suggesting that IAP may have a much broader role in intestinal disorders and in extra-intestinal disorders. Both autophagy [44,45] and IAP [46,47] have been shown in separate studies to control intestinal homeostasis and gut bacteria. In addition, several stimuli that upregulate IAP expression such as vitamin D, zinc, and butyrate in intestinal cells are also activators of autophagy, suggesting a further link between IAP and autophagy. While the discovery of IAP-induced autophagy is new, abundant indirect evidence from other studies and direct evidence from our recent study point to the possibility of a close relationship between IAP and autophagy. 

### 5.1. IAP and Autophagy and Barrier Function

IAP induction of autophagy may explain a novel mechanism by which IAP indirectly maintains barrier integrity and tight junction protein levels. Several studies have highlighted the protective role of autophagy in barrier function. Resveratrol treatment in mice increased TJP levels and improved barrier dysfunction induced by DSS, by inducing autophagy [48]. Inhibition of autophagy by 3-methyladenine (3-MA) exacerbated non-essential amino acid NEAA deprivation-induced barrier dysfunction [49]. Fecal microbial transplant (FMT) was also shown to alleviate gut barrier injury caused by *E. coli* K88 by inducing autophagy [50]. Autophagy was also found to promote barrier function by causing degradation of TJP claudin-2, a mediator of leaky gut [51]. This is parallel to the role of IAP decreasing claudin-2 expression [19]. Caludin-2 has been found to be upregulated in IBD [52] which is associated with defective autophagy and IAP deficiency. Thus, it is possible that the barrier protective functions of IAP may be orchestrated via autophagy. Future studies using gain- and loss- of function of autophagy downstream of IAP will be helpful in examining this possibility.

### 5.2. IAP and Autophagy in Inflammation

IAP is well known for its anti-inflammatory role in the gut, mainly via its direct detoxification of pro-inflammatory molecules such as LPS, and nucleotides such as ATP and UDP [5]. However, an indirect anti-inflammatory role of IAP is also evident by our own observation that showed autophagy-dependent anti-inflammatory function of IAP [10]. IAP requires TLR4 signaling to mediate its anti-inflammatory effects, as IAP ameliorated colitis and inhibited IL-6 and TNF-α production in WT but not in TLR4-KO mice [28]. IAP also induced autophagy in a TLR-4 dependent manner [10], suggesting that IAP may exert its anti-inflammatory effects through autophagy. The anti-inflammatory role of autophagy is evident in CD patients with the autophagy risk allele of Atg16 T330A, who have a higher production of proinflammatory cytokines [53]. Other studies have also highlighted the anti-inflammatory role of autophagy [54,55]. It is vital to understand how IAP may regulate anti-inflammatory autophagy to gain a better understanding of the pathophysiology of the disease and for identifying novel targets to control inflammation.

### 5.3. IAP and Autophagy and Gut Microbes

Connections between IAP, autophagy, and gut microbes can be recognized in many models of intestinal diseases. One such example is sepsis. IAP has been used in preclinical and clinical studies for treating sepsis and sepsis-related conditions such as acute kidney injury [5,56]. The protective role of autophagy in sepsis is also well known [57,58] Additionally, polymorphism in autophagy genes has been linked to the pathogenesis of sepsis [59,60]. It is possible that in the setting of sepsis and possibly other conditions of dysbiosis associated with bacterial translocation, IAP relays information to induce autophagy to activate downstream events such as direct engulfment of resident bacteria in autophagosomes before they infiltrate further in the deeper tissues or to release antimicrobial proteins such as lysozyme in the lumen that destroy the bacteria. Thus, IAP may control gut microbes and restore intestinal homeostasis by inducing downstream antimicrobial autophagy. Whether or not autophagy induced by IAP directly engulfs bacteria, such as commensals and pathogens that may translocate into the intestinal epithelium or to other organs such as lungs in the setting of dysbiosis, remains to be demonstrated.

### 5.4. IAP, Autophagy and Mucin

Mucus is one of the critical arms that maintain the gut barrier integrity. Mucus acts as a physical partition between the intestinal lumen and the epithelial cell layer. Mucus also acts as a scaffold where a high concentration of antimicrobial proteins are found [61,62]. Additionally, mucus has also been shown to encapsulate the bacteria in the colon [63]. The mucus layer is comprised of an intricate arrangement of O-linked glycoproteins called mucins that are produced by goblet cells [62]. In the intestine, the most predominant mucin is Muc2. Defective mucus production has been linked to diseases such as IBD [64,65]. While a direct relationship between IAP and mucus has not been established, a few studies have examined this association. It was found that Western diet (WD)-induced disruption of the mucus layer was attenuated in intestine-specific IAP transgenic mice that overexpress human chimeric IAP (Ldlr^−/−^IAP^Tg^) [11]. It was also shown that colonic IAP activity increased following ingestion of fermentable fibers and this was associated with increased mucin production. Conversely, it was demonstrated that Muc2^−/−^ mice exhibited impaired IAP expression and a reduced LPS detoxification [12]. How these two pathways are linked together remains unknown. A common link between these two pathways is autophagy, as autophagy affects the secretion of mucin by goblet cells [66,67]. Thus, it is possible that a positive correlation between IAP and mucin may exist via autophagy. It will be interesting to study whether IAP induce autophagy to promote mucin secretion to provide a physical barrier between the mucosa and bacteria.

## 6. Inducers of IAP and Autophagy

IAP and autophagy can be upregulated by common inducers such as butyrate, vitamin D, and zinc, all of which have been demonstrated to play an important role in maintenance of intestinal homeostasis.

It is possible that the outcomes observed in the setting of deficiency of these inducers is manifested through deficiency of IAP and autophagy.

### 6.1. Butyrate

Butyrate is a short chain fatty acid produced in the gut by resident microbes, most prominent being *Faecalibacterium prasunitzii*, *Roseburia* spp. and *Eubacterium* spp. [68]. Butyrate has been shown to confer anti-inflammatory effects [69], to maintain barrier function [70], and to regulate expression of TJPs [71,72] mucins and antimicrobials [73,74]^.^ Gene expression of IAP was found to be upregulated by sodium butyrate in the porcine small intestine [75]. IAP activity was also enhanced by butyrate in intestinal epithelial cells [76,77,78]. The main mechanism of butyrate-induced IAP expression has been discovered to be via the inhibition of histone deacetylase [79]. Butyrate also induces autophagy [80,81]. As butyrate acts upstream of IAP and IAP induces autophagy, it is possible that protective effects of butyrate are mediated through IAP via autophagy. Future studies are needed to explore these possibilities. It will also be important to understand whether introduction of butyrate-producing bacteria has any impact on IAP function.

### 6.2. Vitamin D

Vitamin D is acquired from production by skin exposed to sunlight and also from dietary sources. Vitamin D deficiency has been linked to plethora of diseases such as IBD, diabetes, cancer, and autoimmune diseases [82,83,84]. Vitamin D binds to its receptor, vitamin D receptor (VDR), a nuclear receptor and a transcription factor that regulates gene expression of several cellular pathways. In the gut, vitamin D signaling has been shown to enhance barrier function by upregulating the expression of TJPs such as ZO-1 and occludin [85], and to influence gut microbiota [86,87]. Vitamin D was found to increase transcriptional expression of IAP and alkaline phosphatase activity in Caco-2 cells [88]. In vivo, it was shown that a vitamin D-restricted high-fat diet downregulated mRNA expressions of IAP isozymes in the duodenum of the ovariectomized mouse [89]. Similarly, IAP activity was found to decrease in the duodenum of rats that were fed a high-fat diet with vitamin D restriction [89]. Similar to IAP, autophagy is also upregulated by vitamin D [90]. Vitamin D enhanced VDR-mediated expression of Atg16L1mRNA [90]. As vitamin D is essential to all arms of innate immune mechanisms for maintaining intestinal homeostasis, studies are needed to understand the mechanistic relationship between vitamin D, IAP and autophagy.

### 6.3. Zinc

Zinc is an essential trace element involved in various biological processes including maintenance of intestinal homeostasis. Zinc is absorbed in the small intestine and its cellular levels are regulated by zinc transporters [91]. Zinc deficiency has been linked to IBD and other gastrointestinal disorders [92,93] and supplementation with zinc improves the outcomes in CD patients [94] and also has been shown to improve barrier function [95]. Zinc exhibits anti-inflammatory properties, as zinc deficiency was found to be associated with increased IL-1β production in animals treated with LPS [96,97]. The role of zinc on IAP function has also been examined. Zinc deficiency in rats was found to reduce alkaline phosphatase activity including that of IAP [98]. In mice, supplementation with zinc oxide was shown to increase IAP transcript [99]. In addition, piglets fed with zinc exhibited an increase in IAP activity while no effect was observed at gene expression level [100]. Thus, zinc appears to be a vital molecule that enhances IAP functions. Zinc has also been shown in many studies to promote autophagy [101,102]. Understanding whether zinc mediates its protective affects by inducing IAP and autophagy will be important to identify new targets in zinc-related therapies.

## 7. Concluding Remarks

**Figure 1:**
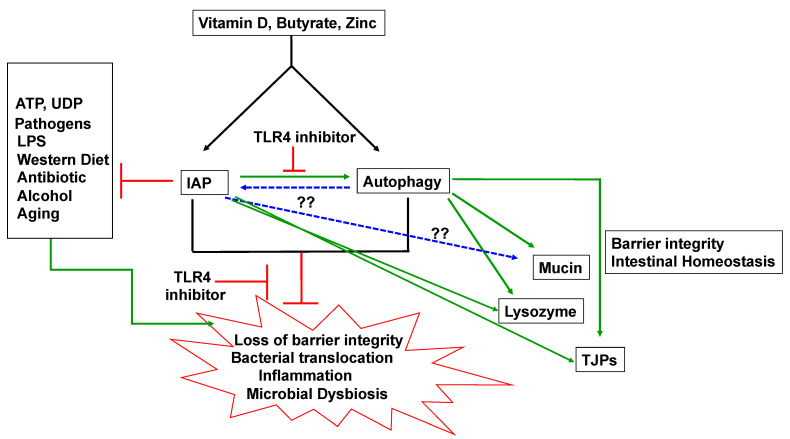
Summary of protective roles of IAP in intestinal homeostasis. Green arrows represent upregulation/enhancement of downstream pathways; red lines represent down regulation/inhibition of downstream pathways. Blue-dotted lines denote hypothetical relationship. Gut bacterial products such as butyrate and dietary products such as vitamin D and zinc induce IAP as well as autophagy. Harmful stimuli such as pathogens, inflammatory molecules and Western diet trigger loss of epithelial barrier integrity and increase inflammation, microbial dysbiosis, and bacterial translocation. IAP inhibits harmful effects caused by these stimuli and protects the gut and maintain homeostasis. Defensive functions of IAP include LPS detoxification, regulation of TJPs, regulation of lysozyme and induction of defensive autophagy. Protective effects of IAP including its induction autophagy are dependent on TLR4 signaling. Autophagy activated by IAP may further promote mucin and antimicrobial protein production, protect barrier function, prevent bacterial translocation and protect against inflammation. Whether or not autophagy has any role in controlling IAP production and whether IAP controls mucin production needs to be explored (blue dotted lines). By inducing autophagy, IAP pairs up with another innate immune mechanism and amplifies its protective functions in the gut. This allows the host to respond to and correct dysbiosis-related effects in the gut and restore homeostasis.

Intestinal alkaline phosphatase (IAP) is a vital innate defense protein involved in maintaining homeostasis. The role of IAP in protecting the gut has been identified at multiple levels. Figure 1 summarizes various defensive roles of IAP in gut homeostasis. These include regulation of gut microbiome, maintenance of epithelial gut barrier via regulation of TJPs, and anti-inflammatory effects of IAP. Given its excellent safety profile, IAP is a promising candidate for potential treatment of many conditions such as atherosclerosis, skin burns, liver fibrosis, autism, and aging. Thus, protective roles of IAP in physiology and pathophysiology appear to be universal and extend far beyond the earlier reported roles in protecting against intestinal disorders. Future pre-clinical and clinical studies are needed to further test IAP for its ability in potentially preventing or treating a myriad of diseases, intestinal and extra-intestinal. Moreover, identification of novel molecular mechanisms of IAP function would identify future therapeutic targets. As IAP induces autophagy, it further provides a new platform for using IAP in understanding diseases that are associated with dysfunctional autophagy such as infectious diseases, neurodegenerative diseases, IBD, and cancer. As data on the link between IAP and autophagy are currently very limited, more studies are needed to further validate and extend these findings in other systems and in clinical studies.
